# Monowave and polywave light-curing of bulk-fill resin composites: degree of conversion and marginal adaptation following thermomechanical aging

**DOI:** 10.1080/26415275.2021.1937181

**Published:** 2021-07-26

**Authors:** Sheila Celia Mondragón Contreras, Ana Luiza Barbosa Jurema, Evaniele Santos Claudino, Eduardo Bresciani, Taciana Marco Ferraz Caneppele

**Affiliations:** Department of Restorative Dentistry, São Paulo State University (UNESP), São Paulo, Brazil

**Keywords:** Bulk-fill, composite resin, degree of conversion, marginal gap, photopolymerization

## Abstract

*Aim:* This study aimed to evaluate the effect of polymerization with either a monowave (MW) or a polywave (PW) light-curing unit (LCU) on the degree of conversion (DC) and marginal adaptation following thermomechanical aging of an ormocer bulk-fill resin composite (RC) (Admira fusion X-tra Bulk Fill – AB), a methacrylate-based bulk-fill RC (Tetric N-Ceram Bulk Fill – TB) and a conventional RC (Tetric N-Ceram – TC).

*Methods:* DC was assessed in five samples of each RC using Fourier transform infrared spectroscopy. For determination of marginal adaptation, standard preparations were made in 60 bovine incisors, divided into three groups, according to the RC. The bulk-fill RC was inserted in a single increment of 4 mm. In contrast, the conventional RC was inserted in three increments. Marginal gap was evaluated after thermomechanical aging. Data were analyzed using a two-way analysis of variance (ANOVA) and Tukey’s tests for multiple comparisons (*α* = 0.05).

*Results:* The two-way ANOVA showed a significant effect (*p*<.05) of the RC factor but not of the LCU factor. The Tukey test showed that TB had the significantly lowest DC followed by TC, and with AB having the significantly highest DC. For the marginal adaptation, a significant effect was found for the LCU factor and the for the interaction RC × LCU (*p*<.05). Groups light-cured with PW showed significantly wider marginal gaps than MW. TC presented wider marginal gaps (17.36 µm) when cured with PW than when cured with MW (13.05 µm). The two bulk-fill RC resulted in similar marginal gap formation to each other.

*Conclusion:* The ormocer-based bulk-fill RC showed a higher DC than the methacrylate-based bulk-fill RC but similar marginal adaptation. The LCU, MW or PW, had no significant influence on the DC, and no relevance on the marginal adaptation.

## 1. Introduction

Resins composites are currently first choice materials for dental restorations because of their high esthetics and acceptable longevity [1]. Manufacturers continuously strive to improve the properties, but there continues to be severe weaknesses related to material handling, technical sensitivity and light curing. These weaknesses can influence physical and mechanical properties that are important for optimal clinical performance [2,3].

The most common clinical problems presented by posterior dental restorations have been related to the polymerization shrinkage stress [4]. Marginal gap formation arises when the polymerization shrinkage is higher than the adhesive bond to the dental tissues [5]. These gaps can lead to marginal infiltration. In association with other factors (diet, oral hygiene, host susceptibility), it may facilitate the further development of caries at the tooth–restoration interface [6], as well as the appearance of clinical signs and symptoms such as marginal discoloration and postoperative sensitivity [7,8]. On the other hand, insufficient polymerization characterized by a low degree of conversion (DC), produce adverse biological reactions and reduce the physical–mechanical properties of the resin composite (RC) [9].

The incremental technique is recognized as a gold standard for conventional RC placement to minimize the issues caused by shrinkage stress [10]. The technique implies the use of horizontal or oblique increments with a maximum layer thickness of 2 mm. Thus, this technique requires longer handling time and increases the risk of contamination and incorporation of voids between layers [11].

The development of RCs to overcome these drawbacks was the impetus for the introduction of the bulk-fill RCs. These RCs can be applied in increments of 4–5 mm. Bulk-fill RCs have increased translucency, new types of photoinitiators, changes in the content of organic and inorganic fillers [12], and may use differentiated monomers (ORMOCER), pure or modified. Conventional RCs based on organically modified ceramics (ORMOCER) have benefits ranging from lower polymerization shrinkage [13] to good wear resistance and an higher elastic modulus compared to other conventional RCs [14,15].

In some bulk-fill RCs, camphorquinone (CQ), which is the most common photoinitiator in conventional RCs, is also used [12]. First and second-generation light-emitting diode (LED) light-curing units (LCUs) show one emission peak (monowave, MW) that matches the absorption spectrum of CQ (430–500 nm) [16]. However, such LCUs may not provide adequate cure of RCs containing alternative initiators. Tetric N-Ceram Bulk Fill (TB) contains Ivocerin, a photoinitiator characterized by high quantum efficiency and high absorption capacity. This germanium-based initiator system has a greater photo-curing activity than CQ. The absorption peak of Ivocerin is set in the violet spectrum (380–420 nm) and slightly extends to the blue spectrum range (420–455 nm), where almost 50% of its peak absorbance occurs at 440 nm. Ivocerin is a photoinitiator with higher photopolymerization reactivity [17]. The third-generation LED LCUs are considered to be broad-spectrum devices. They have two or more emission peaks (polywave, PW) with narrower wavelengths; violet to activate alternative photoinitiators and blue to activate CQ [18].

Considering the introduction of RCs with improved handling (bulk fill RCs) and the use of alternative components (ORMOCER and photoinitiators) in RCs, the influence of the type of LCU (MW vs. PW) on the polymerization efficiency was investigated. Thus, this study aimed to evaluate the effect of curing with an MW or a PW LCU on the DC and marginal adaptation following thermomechanical aging of ormocer and methacrylate-based bulk-fill RCs.

The null hypotheses tested were that there is no significant influence on DC and marginal adaptation of RCs according to: (1) the LCU (MW vs. PW) used for light-activation or (2) type of the RC (ormocer-based bulk-fill RC vs. methacrylate-based bulk-fill RC vs. conventional RC).

## 2. Methods and materials

### 2.1. Light-curing units

Two different LCUs were selected for this study, an MW LED curing unit (3M ESPE, Sumaré, Brazil) and a blue-violet PW LED curing unit (Bluephase N, Ivoclar Vivadent, Schaan, Liechtenstein). The LCUs were used in the high-intensity continuous mode, with a curing time of 20 s. Table 1 presents information about the LCUs. The irradiance (mW/cm^2^), radiant exposure (J/cm^2^) and spectral emission (nm) delivered by the LCUs were checked using a simulation device (Managing Accurate Resin Curing – Patient Simulator (MARC-PS)) (BlueLight Analytics, Halifax, Canada).

**Table 1.** Information about the light-curing units.

Light-cure unit	3M ESPE (3M ESPE, Sumaré, Brazil)	Bluephase N (Ivoclar Vivadent, Schaan, Liechtenstein)
Type	LED 2nd generation monowave	LED 3rd generation polywave
Radiant exposure (J/cm^2^) (mean; standard deviation)	23.48; 0.23	22.99; 0.69
Irradiance (mW/cm²) (mean; standard deviation)	1181.2; 12.7	1145.3; 33.9
Wavelength (nm)	423.87–507.33	381.44–507.33

The radiant exposure (J/cm^2^), irradiance (mW/cm^2^) and wavelength (nm) delivered by the light-curing units were measured using the Managing Accurate Resin Curing – Patient Simulator (MARC-PS).

### 2.2. Restorative materials

The investigated materials included two bulk-fill RCs (TB and Admira Fusion X-tra Bulk Fill (AB)) and a conventional RC (Tetric N-Ceram (TC)). The adhesive system Single Bond Universal was used with all three RCs. Table 2 presents the characteristics of the materials used.

**Table 2.** Manufacturer information for the resin composites and adhesive system used in the study.

Resin composite/adhesive	Shade	Matrix	Filler	w/v%	Manufacturer
Tetric N-Ceram Bulk Fill (TB)	IVA	Bis-GMA, UDMA	Ba-Al-Si glass, YbF3, PPF and mixed oxide, and copolymers	80/55	Ivoclar Vivadent, Schaan, Liechtenstein
Admira fusion X-tra Bulk Fill (AB)	U	Ormocer	Ba-Al-Se-Glass/Silica Nanoparticles	84/69	Voco, Cuxhaven, Germany
Tetric N-Ceram (TC)	A2	Bis-GMA, UDMA	Barium, glass, YbF3, SiO_2_ mixed oxide, eco-polymers	77/53	Ivoclar Vivadent, Schaan, Liechtenstein
Single Bond Universal		MDP phosphate monomer, dimethacrylate resins, HEMA, Vitrebond copolymer	Silica	–	3M ESPE, Sumaré, Brazil

Bis-EMA: bisphenol ethoxylate methacrylate; Bis-GMA: bisphenol glycidyl methacrylate; PPF: pre-photopolymerized particles; TEGDMA: triethylene glycol dimethacrylate; UDMA: urethane dimethacrylate; YbF3: ytterbium trifluoride; w/v%: weight/volume percentage.

### 2.3. Degree of conversion

The DC was measured using Fourier transform infrared spectroscopy (FTIR-ATR, Perkin Elmer, Waltham, MA). The spectra were recorded in real-time before light curing and 15 min after light curing. The FTIR spectrometer was operated under the following conditions: 4000–500 cm^−1^ wavelength, 4 cm^−1^ resolution, and 32 scans. The uncured RC paste was applied directly on the diamond ATR (attenuated total reflection) crystal in a silicon mold of 2 mm in height (the conventional RC) and 4 mm in height (the bulk-fill RCs) corresponding to the maximum increment thicknesses recommended by the manufacturers, making sure that the RC entirely covered the crystal. Samples were cured for 20 s by placing the LCU directly on the top surface of the sample. This procedure was completed for five samples of each of the three resins and two LCUs, resulting in a total of 5 × 3×2 = 30 measurements of the DC. The DC was calculated based on measurements made on the bottom of the uncured and cured samples of the absorbance intensities of the aliphatic C═C peak at 1634 cm^−1^ and that of a standard internal aromatic C═C peak at 1608 cm^−1^ (for the dimethacrylate-based RCs) or 1592 cm^−1^ (for the ormocer-based RC). The DC was calculated as a percentage for each sample using the following equation:

$$\text{Degree of conversion }(\%)=[1-\frac{(1634{\mathrm{cm}}^{-1}/1608{\mathrm{cm}}^{-1}\text{ or }1592{\text{ cm}}^{-1})\mathrm{cured}}{(1634{\mathrm{cm}}^{-1}∕1608{\mathrm{cm}}^{-1}\text{ or }1592{\mathrm{cm}}^{-1})\mathrm{uncured}}]\times 100$$

### 2.4. Marginal adaptation

#### 2.4.1. Sample preparation and distribution

**Figure 1.**
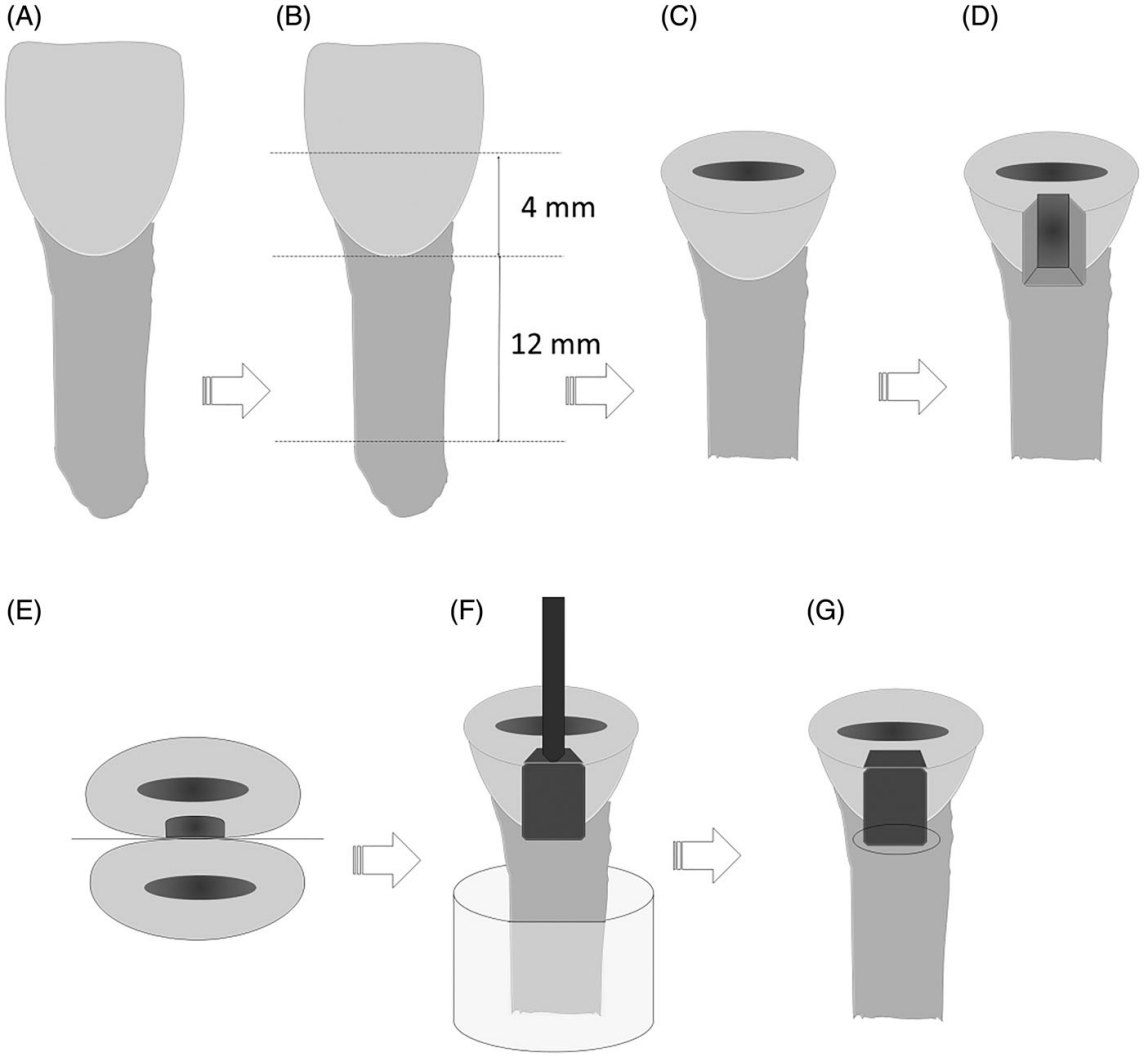
Experimental design. (A) Bovine incisor; (B) sectioning the tooth crown on flat surface 4.0 mm above the CEJ and the root 12.0 mm below the CEJ. (C) Final dimensions of the tooth; (D) class II slot preparation in the proximal and occlusal-gingival directions; (E) positioning with the adjacent tooth and metal matrix for restoration placement; (F) loading device on top of the restoration; (G) marginal gap measurement area.

Sixty extracted bovine incisors were collected, cleaned and stored in a 2% thymol solution. A flat surface on the buccal surface was created by grinding in a polishing device with grit #1200 aluminum oxide abrasive papers (FEPA-P; Struers, Ballerup, Denmark). The incisal surfaces were horizontally sectioned 4.0 mm above the cementoenamel junction (CEJ) and the roots 12 mm below the CEJ with a double-faced diamond disc (KG Sorensen, Barueri, Brazil), allowing for the configuration of a flat standard occlusal surface (Figure 1(A–C)). Standard cavities were made using a high-speed diamond bur (#FG 4138, KG Sorensen, Barueri, Brazil) under continuous water-cooling. Cavities simulated a class II preparation, with cervical margins in dentin. The cavity preparations were 3.0 mm wide, 4.0 mm high and 1.5 mm deep (Figure 1(D)). The burs were replaced after every five preparations.

The cavities were randomly allocated into six distinct groups according to the RC material and LCU. For the restoration, the teeth were fixed in an acrylic mold with an adjacent tooth and a metal matrix strip of less than 5 mm in height (TVD, Santa Catarina, Brazil) (Figure 1(E)). The cavities were dried with a cotton ball. The adhesive system Single Bond Universal was applied following the manufacturer's instructions. The restorative procedure was performed according to the group, as follows:

The bulk-fill RCs were applied to the cavity in a single increment of 4 mm. Half of the bulk-fill restorations were light-cured with the MW LCU for 20 s in high continuous intensity mode (*n* = 10). The other half of the bulk-fill restorations were light-cured using the PW LCU for 20 s in high continuous intensity mode (*n* = 10).

The conventional RC was applied in two oblique and one horizontal increment. Light-curing was performed after the insertion of each increment using the LCUs described above.

#### 2.4.2. Aging by thermomechanical cycling

For artificial aging, the teeth were covered by a simulated periodontal ligament of mercaptan-based material (Permlastic; Kerr, Orange, CA) with a thickness of 0.2–0.3 mm [19]. All samples were submitted to thermomechanical cycling in a thermomechanical wear machine (ER 3700, ERIOS, São Paulo, Brazil) with a 15.0 mm cylindrical metallic tip attached to a steel bar placed in contact with the restoration (Figure 1(F)). The load was applied perpendicularly to the occlusal surface of the restoration. The loading device delivered an intermittent axial force of 88.4 N at two cycles/s, and 120,000 load cycles were applied. Simultaneously, 5000 thermal cycles were performed using water baths at temperatures of 5 (±2 °C), 37 (±2 °C) and 55 (±2 °C) for 30 s at each temperature, with a 10 s interval between each bath.

After thermomechanical aging, the width of marginal gaps at the gingival wall (Figure 1(G)) was measured (µm) using a stereomicroscope (Discovery V20, Zeiss, Gottingen, Germany) at ×50 magnification. Three measurements per sample were made, one near the buccal-gingival line angle, one at the center of the gingival wall and one near the linguo-gingival line angle.

### 2.5. Statistical analysis

The Kolmogorov–Smirnov test confirmed the normality of the data. Two-way analysis of variance (ANOVA) was used to compare the DC and the marginal gap results among the groups, followed by the Tukey test for multiple comparisons. Degree of conversion and marginal gap were correlated using Pearson's correlation. The data were analyzed statistically using the Software Statistica for Windows (Statsoft, Tulsa, OK) at a significance level of 0.05.

## 3. Results

### 3.1. Degree of conversion

**Figure 2.**
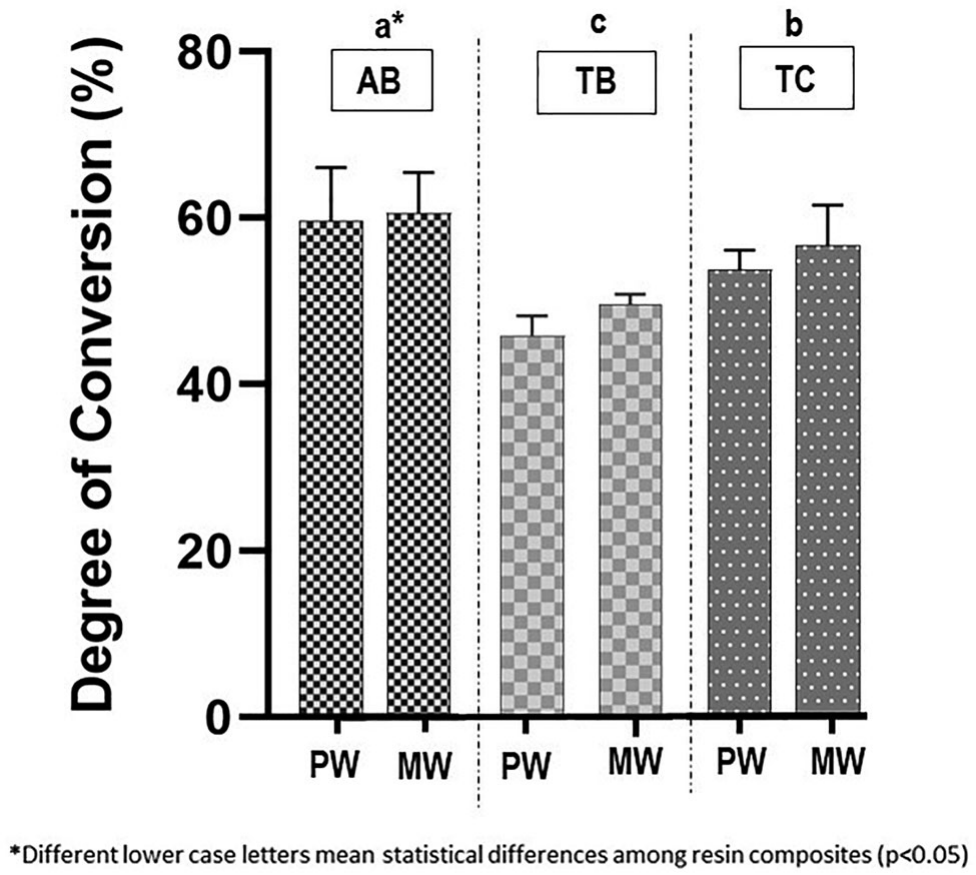
Degree of conversion of the three resin composites for each light-curing unit. PW: polywave; MW: monowave; AB: Admira fusion X-tra Bulk Fill; TB: Tetric N-Ceram Bulk Fill; TC: Tetric N-Ceram.

Two-way ANOVA revealed a significant effect of the RC (*p*<.05) but not of the LCU on DC. The Tukey test subsequently showed that TB (mean value: 47.80%) had the significantly lowest DC followed by TC (mean value: 50.97%) and finally by AB which had the significantly highest value of DC (mean value: 60.11%) (Figure 2).

### 3.2. Marginal adaptation

**Figure 3.**
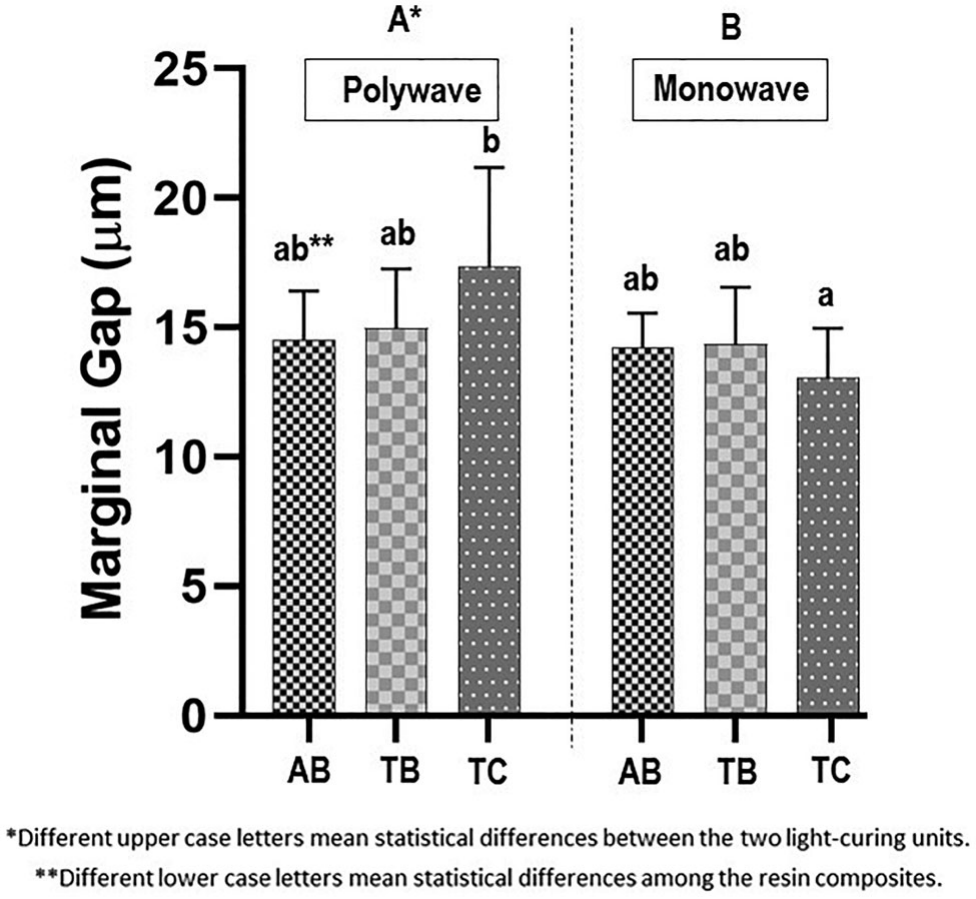
Marginal gap width of the three resin composites for each light-curing unit. AB: Admira fusion X-tra Bulk Fill; TB: Tetric N-Ceram Bulk Fill; TC: Tetric N-Ceram.

The two-way ANOVA found a significant effect of the LCU on marginal gap width after aging (*p*<.01) and a significant interaction between LCU and RC (*p*<.05). The Tukey test subsequently showed that the MW LCU resulted in significantly lower marginal gap width (mean value: 13.87 µm) than did the PW LCU (mean value: 15.62 µm) and that TC light-cured with the PW LCU resulted in a significantly higher marginal gap width (mean value: 17.36 µm) than when light-cured with the MW LCU (mean value: 13.05 µm) (Figure 3).

### 3.3. Pearson’s correlation

Pearson's correlation test showed no statistically significant correlation between the DC and marginal gap for the tested groups (*r*= −0.23; *p*>.05).

## 4. Discussion

According to the results for the DC, the first null hypothesis was accepted. Different LCUs did not affect the DC. The importance of using a PW LCU has been stressed in a clinical scenario when using RCs that present different photoinitiators. According to the results of our study, no difference on conversion was detected regardless the difference in photoinitiators within the tested RCs. The second null hypothesis was rejected. AB showed the highest DC, compared to TB, which had the lowest value. The minimum DC values for clinically acceptable RC restorations have not yet been established. The RC damage caused by abrasive wear is reported to be related to values of DC lower than 55% [9]. These values could represent a minimum DC that can be accepted for adequate restorative material performance. In the present study, the value of the DC of TB (47.8%) was lower than the minimum values acceptable. A more considerable amount of filler particles in conjunction with the high viscosity monomer bisphenol A-glycidyl methacrylate (bis-GMA), present in TB composition, could block the passage of an adequate amount of light sufficient to reach 4 mm depth. The high content of filler particles in TB resin (61% by volume) also explains the decrease in the material's translucency and consequently the low DC, once the DC may be influenced by material composition (matrix and filler) and translucency [20]. A high content of inorganic particles and the irregular shape of the particles, increases the dispersion of light decreasing the transmittance of light [21]. To overcome the low translucency of TB, the manufacturer indicates the uses of an additional photoinitiator system based on dibenzoyl germanium, called ‘Ivocerin’ excited by light between 380 and 450 nm and which has a higher activity than the CQ, due to its more significant absorption of visible light [22]. However, in this study, even with this new TB component, the DC did not increase. This low conversion could be related to low penetration of short-wavelength (violet) light within the RC. Increasing the irradiation time to achieve light penetration in deeper layers could increase conversion by using a higher energy density, approximately 47.03 J/cm^2^, to obtain the minimum DC required for a greater thickness [23]. More studies on longer light activation are necessary to properly answer that question.

On the other hand, bis-GMA free bulk-fill RC, based purely on ORMOCER, a three-dimensionally cross-linked inorganic–organic polymers with high filler content (84% by weight), achieved a DC value higher than TB. The ormocer molecule, contained in the AB, presents the alkoxysilyl groups of silane in its composition that allows the formation of an inorganic link of Si–O–Si by hydrolysis and polycondensation reactions. The reported link formation would significantly improve the DC, even in the presence of a great amount of filler particles in their composition, fact that hinders the passage of light.

Regarding the marginal adaptation, the first and the second null hypothesis were rejected. The LCUs and the interaction of factors (LCU and RCs) affected the external marginal adaptation after thermomechanical aging (*p*<.05).

One of the most common criteria for assessing restorations is whether the marginal interface is intact. In our study, the restorations were submitted to thermomechanical aging prior to assessing the marginal adaptation [24,25]. Thermomechanical aging was used to simulate the degradation of the interface tooth–restoration over time in the oral cavity. The effectiveness of thermocycling as a clinical aging simulator has been controversial [26,27]. Besides, no evidence of the number of cycles that are likely to be experienced *in vivo* has been found. Still, an interim estimate of approximately 10,000 cycles per year has been suggested [28]. In our study, 5000 cycles were applied to the samples.

In general, marginal gaps in the investigated materials were between 13.53 µm and 15.33 µm. Gaps of this magnitude have been considered acceptable and are not considered a failure [28–30]. Despite the differences between groups, this study demonstrated that all RCs under investigation showed satisfactory marginal adaptation after thermo-mechanical aging. More prolonged aging simulation might reveal clinically relevant differences among the tested materials and grant future studies to answer that question.

No correlation was found between the DC and marginal adaptation. The formation of marginal gaps in RCs restoration is very complex. It involves several factors, such as interface stress during light-curing [7], quality of bonding [31], linear coefficient of thermal expansion [32], mastication loading over the restoration [33] and the quality of cure [9].

## 5. Conclusions

The ormocer-based bulk-fill RC showed a higher DC than the methacrylate-based bulk-fill RC but similar marginal adaptation. The LCU, MW or PW, had no significant influence on the DC, and no relevance on the marginal adaptation.
